# Comparative analysis of oral-gut microbiota between captive and wild long-tailed macaque in Thailand

**DOI:** 10.1038/s41598-021-93779-4

**Published:** 2021-07-12

**Authors:** Vorthon Sawaswong, Kesmanee Praianantathavorn, Prangwalai Chanchaem, Ariya Khamwut, Taratorn Kemthong, Yuzuru Hamada, Suchinda Malaivijitnond, Sunchai Payungporn

**Affiliations:** 1 Program in Bioinformatics and Computational Biology, Graduate School, Chulalongkorn University, Bangkok, 10330 Thailand; 2 Research Unit of Systems Microbiology, Chulalongkorn University, Bangkok, 10330 Thailand; 3 Department of Biochemistry, Faculty of Medicine, Chulalongkorn University, Bangkok, 10330 Thailand; 4 National Primate Research Center of Thailand, Chulalongkorn University, Saraburi, 18110 Thailand; 5 Evolutionary Morphology Section, Primate Research Institute, Kyoto University, Aichi, Japan; 6 Department of Biology, Faculty of Science, Chulalongkorn University, Bangkok, 10330 Thailand

**Keywords:** Metagenomics, Microbiome, Zoology

## Abstract

Long-tailed macaques (*Macaca fascicularis*), distributed in Southeast Asia, are generally used in biomedical research. At present, the expansion of human communities overlapping of macaques’ natural habitat causes human-macaque conflicts. To mitigate this problem in Thailand, the National Primate Research Center of Thailand, Chulalongkorn University (NPRCT-CU), was granted the permit to catch the surplus wild-born macaques and transfer them to the center. Based on the fact that the diets provided and the captive environments were different, their oral-gut microbiota should be altered. Thus, we investigated and compared the oral and fecal microbiome between wild-born macaques that lived in the natural habitats and those transferred to and reared in the NPRCT-CU for 1 year. The results from 16S rRNA high-throughput sequencing showed that the captive macaques had distinct oral-gut microbiota profiles and lower bacterial richness compared to those in wild macaques. The gut of wild macaques was dominated by Firmicutes which is probably associated with lipid absorption and storage. These results implicated the effects of captivity conditions on the microbiome that might contribute to crucial metabolic functions. Our study should be applied to the animal health care program, with respect to microbial functions, for non-human primates.

## Introduction

Over the last decade, the study of the microbiome in the digestive system has received dramatically increasing attention^1^. The gut microbiome composition varies among different species, contributing the specific functions to their host^2^. Recent studies have reported that the normal gut microbiota has been implicated in beneficial functions including dietary metabolism, vitamin synthesis, modulating gut mucosal integrity, immunomodulation, and inhibition of pathogen infections^3,4^. Some gut microbes also provide the essential roles for non-digestible carbohydrate fermentation producing the short-chain fatty acids (SCFAs) which are critically important to host health, involving energy harvest, glucose homeostasis and promoting the mucosal immune system^3,5^. Hence, the dysregulation of gut microbiota is associated with many diseases such as obesity^6^, diabetes^7^, inflammatory bowel disease (IBD)^8^, and gastrointestinal cancer^9^. The gut microbiota can be affected by both environmental factors and host factors. However, environmental factors such as habitats, diets, and antibiotic usage, seem to contribute larger effects on gut microbial compositions^10^. The oral microbiota, which is generally composed of saccharolytic and/or proteolytic bacteria, can be altered by several factors such as oral hygiene, diet sources, or foreign invasion^11^. These are both transient and commensal populations which typically form the biofilm on a soft and hard surface in oral cavity^12^. These oral symbionts have been suggested to contribute to the functions of maintaining the oral homeostasis and inhibiting the invasion of pathobionts^13^.

Previously, several animal models have been utilized in functional studies, for a better understanding of the roles of these microbes^14^. The non-human primates (NHPs) were suggested to be the best representative model for humans due to their similar genetic and physiological characteristics^15^. In addition, the NHPs have typically been used in biomedical research, particularly in pharmaceutical development^16,17^. Recent studies have suggested that the microbiota also contributes in the modulation of vaccine and drug responses^18,19^. Accordingly, to utilize the NHPs either in microbiome study or drug and vaccine testing, the baseline microbial profiles of these animals should first be explored.

The long-tailed macaque (*Macaca fascicularis*), also known as cynomolgus macaque, is one of the NHPs commonly used in biomedical research^18,20^, notably in the recent COVID-19 vaccine research^21^. It is widely distributed in Southeast Asian countries^22^ and can be found in all regions of Thailand. Regarding their overpopulation status which has caused the human-macaque conflict^23^, the National Primate Research Center of Thailand, Chulalongkorn University (NPRCT-CU) was granted the permit from the Department of the National Parks, Wildlife and Plant Conservation to catch the wild-borne macaques and use them as breeding founders of the center. These macaques were reared under a well-controlled and hygienic condition and the NPRCT-CU has been awarded the AAALAC International Accreditation since February 2020. Nevertheless, the captivity restricted the roaming and foraging behaviors of captive macaques. In addition, rearing them in the cage may enhance the close contact among macaques and allow the transmission of microbes via oral-fecal route which could affect their microbiome. The shift of diets and the captive living environment could also cause the alteration in their microbiota, as previously observed^24^. A recent study suggested that the microbiota in wild animals could enhance the host response in inflammatory stimuli and improve disease resistance^25^. Concordantly, several primate centers have an incidence of idiopathic diarrhea which was possibly caused by dysbiosis in microbiota^26^. Thus, the studies of microbiome in captive (wild-born) macaques living in NPRCT-CU might elucidate the captivity effects on microbiota composition and their roles in influencing the health of animals. This study aimed to explore the oral and fecal microbiome of the captive macaques compared to those in the wild. The knowledge of this study should be beneficial for translation to animal healthcare and management in the primate centers.

## Results

### Characteristics of macaque and sequencing summary

The characteristics of long-tailed macaques were shown in Table 1. The sex ratio between the two populations had no difference, but the difference was detected for the age which was due to the uncontrollable limitation of subject recruitment. The wild group had significantly older age (*P* = 4.0e−3) and heavier bodyweight (*P* = 0.01) than the captive macaques (tested by *t*-test, *P* < 0.05). The age, sex and body mass sightly influent the beta diversity (Jaccard dissimilarity) of microbiome as showed in Supplementary Fig. S1, but these effects were less than the effect of living condition (wild versus captive). Thus, the comparison of microbiome between wild and captive macaques was the main focus in this study.

**Table 1:** Characteristics of captive and wild macaque populations in this study.

Populations	Age (years)^a^	Bodyweight (kg)^a^	Sex (%)^b^	
			M	F
Captive (n = 43)	3.34 ± 1.67	3.13 ± 1.22	20 (46.5%)	23 (53.5%)
Wild (n = 37)	5.92 ± 4.44 ^c^	4.16 ± 1.93^c^	22 (59.5%)	15 (40.5%)
*P* value	0.004	0.012	0.27	

^a^Values shown as ﻿mean ± SD with* P* value from statistical *t*-test.

^b^Percentage (%) of sex proportion per group tested by Fisher’s exact test.

^c^The absent data of six wild macaques were excluded.

**Figure 1:**
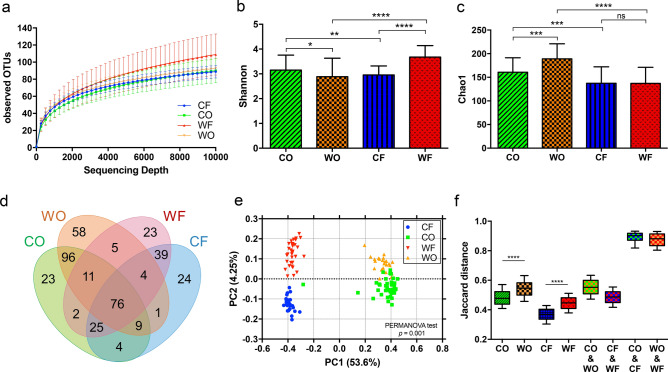
The bacterial diversity in oral-fecal microbiome of wild and captive long-tailed macaques. (**a**) Rarefaction curve indicated the observed OTUs (mean ± SD) against sequencing depth in each group: *CO* captive-oral, *WO* wild-oral, *CF* captive-fecal, *WF* wild-fecal. The alpha diversity was determined by two indices: (**b**) Shannon’s diversity index and (**c**) Chao1 index which were plotted by mean ± SD and statistically tested by Mann–Whitney U test (*P < 0.05, **P < 0.01, ***P < 0.001, **** *P* < 0.0001, *ns* not significant). (**d**) The Venn diagram showed the numbers of shared taxa among groups. (**e**) The beta diversity was plotted in principal coordinate analysis (PCoA) plots based on Jaccard dissimilarity (tested by PERMANOVA analysis). (**f**) The box plot represented the Jaccard distances between communities which were statistically tested by Mann–Whitney U test.

The number of samples and the sequencing output summary per group including raw reads, filtered reads and identified reads were described in Table 2. The samples which were unable to amplify for 16S library preparation were excluded from the study. Over 99% of cleaned reads were successfully classified by the QIIME2 pipeline^27^. The rarefaction analysis was also examined to check the sampling coverage by sequencing depth. The results indicated that the bacterial classification was rarified in all study groups as shown in Fig. 1a.

**Table 2:** Sequencing and reads classification summary.

Groups	n	Raw reads^a^	Filtered reads^a^	% Identified reads^a^
CF	35	95,113 ± 56,460	41,946 ± 27,835	99.49 ± 0.69
CO	32	45,218 ± 13,532	45,028 ± 13,447	99.93 ± 0.05
WF	27	25,905 ± 11,499	25,807 ± 11,468	99.70 ± 0.29
WO	25	73,325 ± 31,141	26,613 ± 12,004	99.49 ± 0.54

The abbreviation represented the macaque groups and sample types including, *CF* captive-fecal, *CO* captive-oral, *WF* wild-fecal, and *WO* wild-oral.

^a^Number of reads (mean ± SD).

### Alpha diversity of oral and fecal bacteria

Bacterial diversity was evaluated based on Shannon’s diversity index (Fig. 1b) while the richness of bacterial operational taxonomic units (OTUs) was determined by Chao1 index (Fig. 1c). The statistical comparisons of indices between groups were carried out by Mann–Whitney U test with *P* < 0.05.

The Shannon’s diversity of oral microbiome in captive (CO) macaques (3.15 ± 0.60) exhibited significantly higher (*P* = 0.03) compared to those in the wild (WO) group (2.88 ± 0.75). However, the Chao1 index indicated the greater OTUs richness (*P* = 8.63e−4) in WO (189 ± 31.81) than the CO (160.8 ± 30.49). The Shannon’s diversity of fecal microbiome in wild (WF) macaques (3.68 ± 0.46) was significantly higher (*P* = 2.41e−8) than the fecal microbiome of the captive (CF) group (2.96 ± 0.36) whereas the Chao1 richness was not significantly different (*P* = 0.92) between WF (137.2 ± 34.1) and CF (137.5 ± 34.7) group. Between oral and fecal microbiota, the Shannon diversity of CO was slightly higher (*P* = 5.64e−3) than CF samples but it was significantly lower (*P* = 2.61e−5) in WO compared to WF. We found significantly higher Chao1 richness in the oral microbiome than the fecal microbiome in both wild group (*P* = 1.15e−6) and captive groups (*P* = 1.03e−4).

To investigate the shared OTUs between groups, the shared OTUs were counted and illustrated in the Venn Euler diagram shown in Fig. 1d. The results showed that about 76 OTUs were shared across all groups suggesting that they constitute the common bacterial populations in the oral-gut environment. Interestingly, 192 sharing OTUs between WO and CO were found, whereas fewer overlapping taxa of WF and CF were noted (comprising only 144 common OTUs). The WO microbiome contained the highest number of unique taxa compared to other groups.

### Beta diversity of microbiota in long-tailed macaque

The beta diversity was investigated based on Jaccard dissimilarity index and presented by principal coordinate analysis (PCoA) plot in Fig. 1e. The distance of centroids and dispersion of the groups were statistically evaluated by permutational multivariate analysis of variance (PERMANOVA) with *P* < 0.05. The findings indicated that the bacterial profiles between wild and captive macaques were significantly different (*P* = 9.99e−4). It also showed a very high dissimilarity (*P* = 2.00e−3) between the oral and fecal microbiome. The Fig. 1f presented the comparisons of average distance between groups and within-group which were statistically tested by Mann–Whitney U test, *P* < 0.05. The result presented significantly higher community distances (*P* = 8.76e−37) in WO group compared to those in CO group while they were also higher (*P* = 5.48e−87) in WF than those in CF microbiome.

### Relative abundance of bacteria phyla

**Figure 2:**
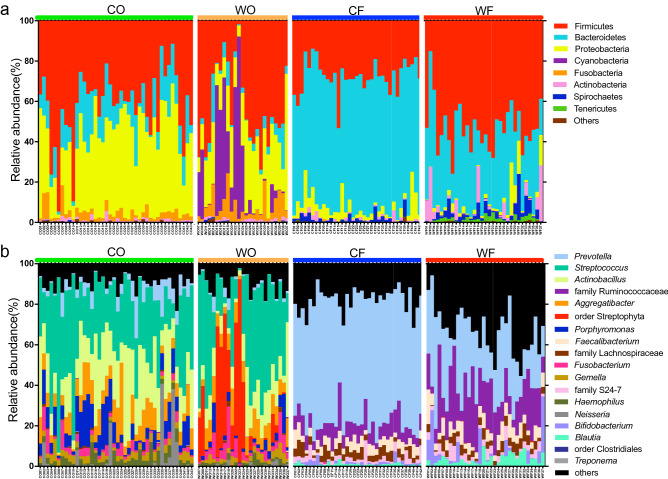
Relative abundance of bacteria in wild and captive long-tailed macaques from 16S microbiome sequencing. The bar plots showed the relative abundance of (**a**) bacterial phylum and (**b**) bacterial genera of classified taxa which were classified by Qiime2 pipeline (*CO* captive-oral, *WO* wild-oral, *CF* captive-fecal, *WF* wild-fecal).

The oral microbiome was dominated by Firmicute bacteria, contributing the proportion (mean ± SD) of 42.1 ± 17.2% and 43.2 ± 19.6% in captive (CO) and wild (WO) macaques, respectively (Fig. 2a). The second most enriched phylum was Proteobacteria which accounted for 38.7 ± 14.0% in CO and 24.6 ± 13.0% in the WO group. The Bacteroidetes was the third most abundant phylum in CO (14.0 ± 8.4%), while Cyanobacteria was the third most enriched bacteria in WO (18.9 ± 25.5%). It was observed that some of WO have a distinctively high abundance of Cyanobacteria. The remaining community included less abundant phyla such as Fusobacteria, Actinobacteria, Spirochaetes, and Tenericutes. The comparisons of phylum tested by Mann–Whitney U test (*P* < 0.05) showed that relative abundances of Bacteroidetes and Proteobacteria were significantly greater in the CO than in the WO (*P* = 1.51e−11 and *P* = 1.08e−3, respectively) while Cyanobacteria was significantly higher (*P* = 1.80e−5) in WO.

The most dominant phyla in the fecal microbiome were Bacteroidetes and Firmicutes. This finding shows that the captive (CF) macaques have a significantly higher abundance (*P* = 4.20e−14) of Bacteroidetes (64.1 ± 6.8%) than wild (WF) macaques (34.4 ± 11.9%). In contrast, the Firmicutes contribute a significantly greater abundance (*P* = 6.16e−6) in WF (52.8 ± 11.5%) than in the CF (27.6 ± 7.8%). The significant difference in phyla abundance also indicated that the CF has greater proportions of Proteobacteria (*P* = 1.31e−12) but lower levels of Tenericutes (*P* = 3.53e−11) than the WF group.

### Dominant taxa in oral and fecal microbiome

To observe the core structure of the bacterial community in both groups of macaques, the top 18 most dominant taxa were identified and presented in Fig. 2b. These results indicate the distinct major population of microbial profiles between groups. *Streptococcus* was the most abundant oral bacteria, contributing the proportion of 31.3 ± 17.2% and 36.1 ± 19.6% in CO and WO, respectively. The other predominant bacteria in the oral microbiome were *Actinobacillus*, *Aggregatibacter*, *Porphyromonas*, *Fusobacterium*, *Gamella*, and *Haemophilus*. The proportion of these bacteria were varied among samples and different between wild and captive groups. The major population of the fecal microbiome in captive (CF) macaques was *Prevotella* from phylum Bacteroidetes, which made up to 60.5 ± 7.7% of bacterial abundance while it accounted for only 27.4 ± 14.4% in the WF group. The remaining enriched fecal bacteria included the family Ruminococcaceae, *Faecalibacterium*, family Lachnospiraceae, family S24-7, *Bifidobacterium*, and *Blautia*.

### Differential abundance analysis of wild and captive macaque

**Figure 3:**
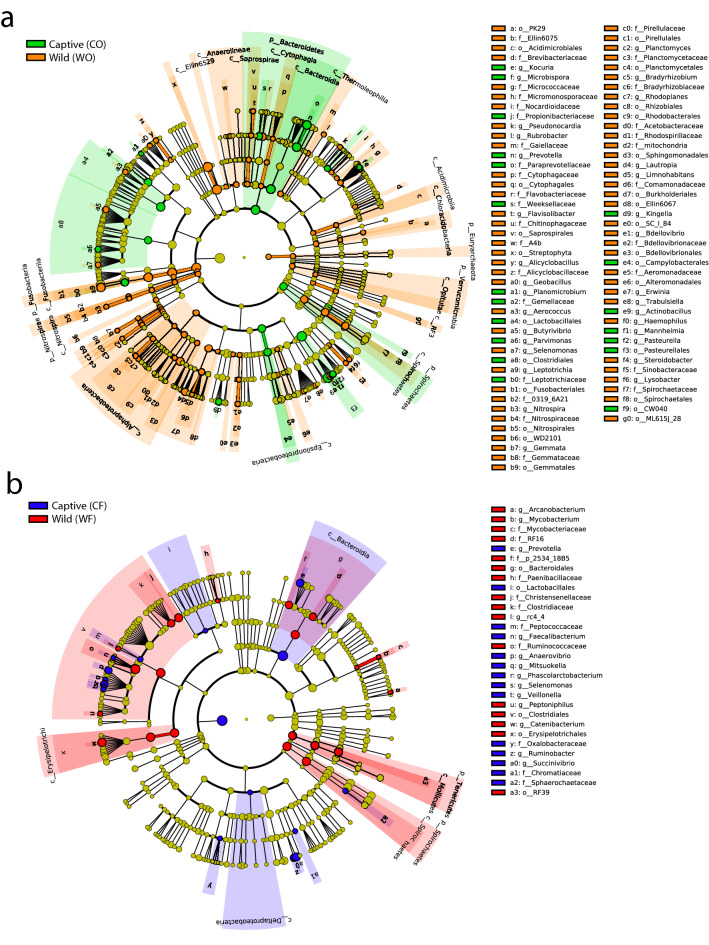
Differential abundance analysis comparing the taxa within wild and captive macaques. The cladogram illustrated the results from Linear discriminant analysis Effect Size (LEfSe) analysis showing the significantly differential taxa (LDA score > 3, *P* < 0.01) in (**a**) Oral microbiome and (**b**) Fecal microbiome. The circle nodes represented the classified taxa and their taxonomic relationship. From the center to the periphery of cladogram, each level represented the taxonomic rank, with phylum, class (c), order (o), family (f) and genus (g), respectively. The size of node proportionally indicated the relative abundance of each taxon. The taxa with no significant differences were represented by yellow color. The other significant different taxa enriched in each group were illustrated by different colors (green = CO, orange = WO, blue = CF, red = WF).

The differential abundance analysis was performed using the LDA Effective Size (LEfSe) tool^28^. The significant taxa with LDA score > 3 and *P* < 0.01 were illustrated in Fig. 3. The oral microbiome of wild (WO) macaques had a greater number of significantly enriched taxa than the captive (CO) macaques, as presented in Fig. 3a. The phylum Bacteroidetes with a high proportion of *Prevotella* and phylum Spirochaetes were more enriched in the CO microbiome. In addition, the CO group also included high levels of bacteria in the Lactobacillales order, Clostridiales, Campylobaterales, and Pasteurellales. In contrast, the most prominent phyla in WO were Verrucomicrobia, Nitrospirae, and Fusobacteria. The various bacteria in the Alphaproteobacteria class were also more abundant than in the CO. As shown in Fig. 3b, comparing the fecal microbial community, revealed that the fecal microbial community has a lower number of significant taxa than the oral community. We identified the enriched bacteria in CF which were mainly *Prevotella, Ruminobacter*, *Succinivibrio,* and order Lactobacillales. In the WF samples, Spirochaetes and Tenericutes were significantly abundant phyla. A significantly greater abundance of *Catenibacterium*, family Ruminococcaceae and family Clostridiaceae were observed in the WF microbiome.

### Functional inference of microbiome in wild and captive macaques

**Figure 4:**
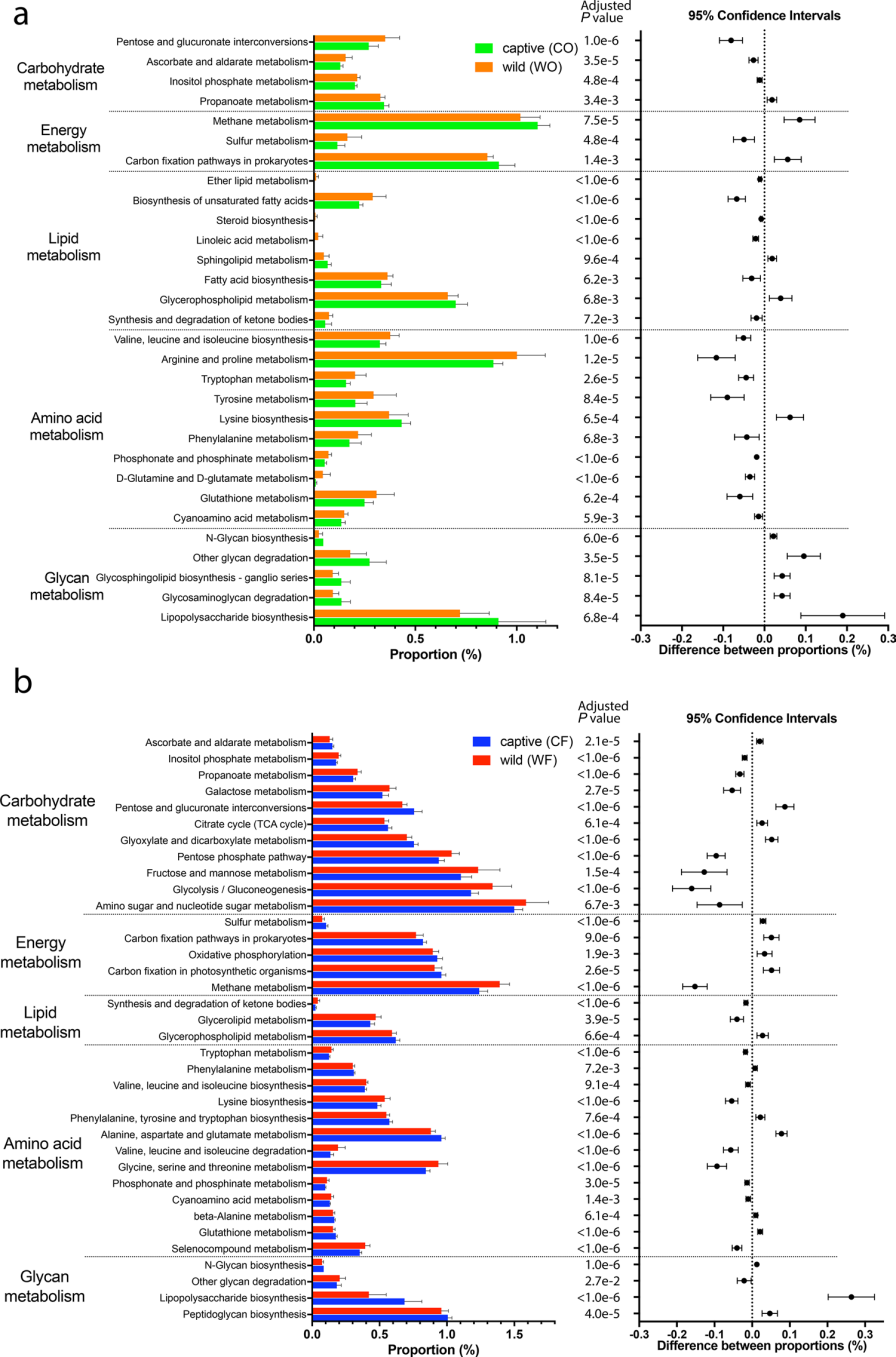
Functional pathway inference of oral and fecal microbiota in wild and captive long-tailed macaques. The significant differential KEGG ortholog (KO) pathways in (**a**) oral microbiome and (**b**) Fecal microbiome of wild and captive macaques analyzed by PICRUSt2 (statistically tested by multiple *t*-test with 1% false discovery rate (FDR) correction). The graph on the left shows the pathway abundances while the right shows 95% confidence intervals of differential abundance.

The functional analysis was carried out using PICRUSt2^29^ following the standard pipeline implemented in QIIME2^27^. Differential pathway abundance comparison was statistically tested by multiple *t*-test (*P* < 0.05) with 1% false discovery rate (FDR) correction. The significant differences of KEGG ortholog (KO) pathway abundance in the oral microbiome were shown in Fig. 4a. The pathway enriched in the WO group is mostly involved with amino acid metabolism while that of the CO group was significantly high in the glycan metabolism. The WO microbiome was also enriched with pathways in lipid metabolism, particularly such as the fatty acid biosynthesis pathway and synthesis and degradation of ketone bodies. The differential functional pathways of the fecal microbiome (Fig. 4b) showed that most pathways related to energy metabolism were enriched in CF macaques. In contrast, the WF group had high abundance of carbohydrate metabolism including pentose phosphate pathway, fructose and mannose metabolism, galactose metabolism, propanoate metabolism, and glycolysis/gluconeogenesis. Moreover, the WF group also had higher lipid metabolism such as synthesis/degradation of ketone bodies and glycerolipid metabolism than in the CF macaques.

### The relationship of Firmicutes/Bacteroidetes ratio and body fat

**Figure 5:**
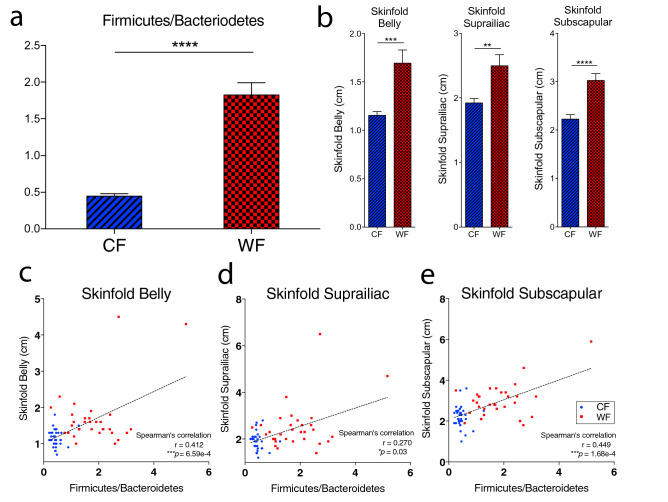
The relationship of Firmicutes/Bacteroidetes ratio in fecal microbiome and body fat accumulations in long-tailed macaque. (**a**) The difference of Firmicutes/Bacteroidetes ratio (mean ± SEM) between wild and captive was compared by *t*-test. (*P < 0.05, **P < 0.01, ***P < 0.001, ****P < 0.0001). (**b**) The bar graph shows the measurement of skinfold indicated body fat (mean ± SEM) in wild and captive macaques compared by *t*-test. The scatter dot plot presents the correlation between Firmicutes/Bacteroidetes and (**c**) skinfold belly, (**d**) skinfold suprailiac, and (**e**) skinfold subscapular analyzed by Spearman’s correlation test.

The Firmicute/Bacteroidetes ratio in fecal microbiome of wild and captive macaques were also compared by *t*-test with *P* < 0.05. The wild macaques exhibit the significant higher Firmicute/Bacteroidetes ratio (*P* = 4.64e−12) as shown in Fig. 5a. The average Firmicute/Bacteroidetes ratios (mean ± SD) were 1.82 ± 0.94 for wild macaques and 0.45 ± 0.19 for captive macaques. The proportion of Firmicute and Bacteroidetes is usually associated with lipid metabolism. Therefore, the skinfolds measured from 3 body sites (belly, suprailiac and subscapular) were analyzed to investigate the relationship between body fat and Firmicute/Bacteroidetes ratio. The skinfold belly, suprailiac and subscapular of wild macaques were significantly higher than those in the captive group (*t*-test, *P* < 0.05) as shown in Fig. 5b. The skinfolds of belly and suprailiac (mean ± SD) of wild macaques were marginally higher than the captive macaques (belly: 1.7 ± 0.77 vs. 1.2 ± 0.23 with *P* = 2.26e−4, and suprailiac: 2.50 ± 0.97 vs. 1.92 ± 0.40 with *P* = 2.20e−3). However, the skinfold subscapular in wild group (3.03 ± 0.80) was significantly greater (*P* = 8.94e−6) than the captive group (2.23 ± 0.52). The correlation between Firmicutes/Bacteroidetes and skinfold was analyzed by Spearman’s correlation test with *P* < 0.05 and showed the significance; skinfold belly (r = 0.41, *P* = 6.59e−4), skinfold suprailiac (r = 0.27, *P* = 0.03), and skinfold subscapular (r = 0.50, *P* = 1.68e−4), as demonstrated in Fig. 5c–e. The association of Firmicutes/Bacteroidetes ratio in oral microbiome and body fat measured by skinfold data was additionally carried out. The result was concordant to that observed in fecal microbiota as shown in Supplementary Fig. S3.

## Discussion

The comparison of microbiome between wild and captive long-tailed macaques presents the perspective knowledge for exploring the bacterial diversity as well as the effects of habitat and captive environments on their oral and fecal microbiota. In this study, the wild macaques showed a greater richness of oral bacteria than the captive macaques, suggesting the higher microbial exposure in their natural habitat. However, the oral microbiota of both macaques was similarly dominated by *Streptococcus* which is the most common bacteria in oral healthy macaques^11,30^. The *Streptococci* group is composed of various species that contribute to different roles such as carbohydrate metabolizer, acid–base regulator, and inhibitor of pathogenic species^31^. These complex species typically colonize in different sites in the oral cavity depending on their specific niches^30^. Other common oral bacteria identified in these macaques were *Actinobacillus*, *Aggregatibacter*, *Porphyromonas* and *Fusobacterium,* some of which are pathogenic to humans^30,32^. However, they are frequently observed in oral microbiota of animals such as chimpanzee and dog^33,34^. Thus, they may not cause diseases to animals, but further investigation is needed to confirm their pathogenesis abilities.

Our study revealed the different abundance of oral microbes between wild and captive macaques. The Bacteroidetes particularly *Prevotella* spp. and order Lactobacillales were more abundant in the captive macaque. Another large group of the order Pasteurellales including *Actinobacillus*, *Aggregatibacter*, *Haemophilus*, and *Pasteurella* were also more enriched in the captive group*.* The order Pasteurellales are normally commensal microbes on mucous membranes of the upper respiratory tract^35^. They also had been recently identified in oral microbiota of captive baboons^36^. In contrast, the oral microbiota of wild macaques was enriched by the group of environmental bacteria such as phylum Verrucomicrobia, phylum Nitrospirae, *Fusobacteria* and various members of class Alphaproteobacteria. These were possibly influenced by the environmental exposure allowing the wild macaque to obtain more natural microbes^37^. For example, *Nitrospira* in phylum Nitrospirae was known as aerobic chemolithoautotrophic which was usually associated with ammonia-oxidization which mainly found in soils, groundwater, rhizosphere and surface of the plants^38^. Various enriched Alphaproteobacteria in wild macaques such as *Gemmata*^39^, Planctomyces^40^, *Bradyrhizobium*^41^, Rhodospirillaceae^42^, and *Limnohabitans*^43^ were typical bacteria found in soil or freshwater which were mostly autotrophs or symbionts in the rhizosphere or bacterioplanktons. There was no evidence supported that these bacteria can be colonized on the epithelia of oral cavity. A recent publication suggested that the permanent colonizers require the specific environment (such as suitable pH and O_2_ level), binding to the host receptor, and crosstalk with other colonizers^30^. Hence, it was hypothesized that several mentioned bacteria were passing microbes which could not colonize and may not be survived in the oral environment.

Since the oral cavity is the entry point of microbial acquisition from the environment, the richness of the oral microbiome was higher than those in fecal microbiota. The limited taxa in fecal microbes might be explained by the selection of the gut environment^44^. Our findings highlighted the lower Shannon diversity of fecal microbiome in captive macaques as a result of the *Prevotella* enrichment. Nevertheless, the fecal microbiota of both groups shared some common characteristics with the gut microbiome of humans and other NHPs^11,45,46^. Therefore, the long-tailed macaque might be a suitable model for investigating the complex interaction and important physiological functions of these gut microbes.

Following the described results, the fecal microbiome of captive macaques was enriched with *Prevotella*, one of the most predominant genera in the human gut which is also a key feature for the classification of three human enterotypes^47^. A previous study also reported that the captive environment which shifted the diversity and type of diet to polysaccharides can cause the transition of primate microbiome to be dominated with *Prevotella*^45^. This had also been observed in other captive mammals^24^. The *Prevotella* spp. was associated with the consumption of high-fiber diets and plant-based carbohydrates especially those found in fruits^48^. Even though all captive macaques were healthy, enrichment of *Prevotella* spp. could increase host susceptibility to mucosal inflammation as reported previously^49^. This raised an interesting point of concern about the effect of dietary change in captive macaques on animal health. Another predominant genus in captive macaques was *Succinivibrio* which was also related to the high fiber and complex carbohydrates intake which resembled that of the *Prevotella* and *Treponema*^50^. Interestingly, the enrichment of order Lactobacillales and *Lactobacillus* spp. in the captive group may be related to the promoting of bile salt hydrolase activity as well as lactic acid and simple sugar fermentation^51^. In addition, they might contribute to the role in the recycling of urea nitrogen that could facilitate the maintenance of the bodyweight of animals fed with high-fiber and low protein diets^52^.

In contrast, the gut microbiota of wild macaques was mainly dominated by several phyla such as Tenericutes, Spirochaetes and Firmicutes, including especially family Ruminococcaceae and *Catenebacterium.* The phylum Tenericutes identified in wild macaques mainly in order RF39 associated with fiber digestion as reported in swine^53^. Similarly, Spirochaetes such as *Treponema* also have cellulolytic capabilities commonly identified in termites^54^ and the rumen of grass-fed animals^55^. The higher abundance of family Ruminococcaceae in wild macaques was related to fibrolytic activity in the gut^56^. The members of the family Ruminococcaceae such as *Fecalibacterium prausnitzii* are known to be a butyrate-producing microorganism that enhances anti-inflammatory effects in the mucosal environment^57^. Depletion *of F. prausnitzii* was known to be associated with inflammatory bowel disease^58^. The *Catenibacterium,* another abundant genus in wild macaques in this study, was known to produce the short-chain fatty acids (SCFAs) including acetate, butyrate, and lactate from carbohydrate fermentation that may extract a high yield of energy from carbohydrate-rich diets for the host^59^. The increase in Catenibacteria could induce the energy storage and obesity in humans and mice^60^.

These findings imply that both groups of macaques mainly consumed high-fiber diets supporting the observation that both groups of animals took various kinds of tropical fruits. Basically, the wild macaques foraged for some seasonal fruits in their habitats such as mangoes, figs and bananas while the captive macaques were fed with monkey pellet and fresh fruits. The distinct sources and types of these diets may differently affect the gut microbiota causing the alteration of energy usage and storage.

Generally, the abundance of phylum Firmicutes in the fecal microbiome is known to be associated with fatty acid absorption and lipid metabolism^61^. Interestingly, wild long-tailed macaques were significantly enriched by Firmicutes, so the Firmicute/Bacteroidetes ratio was higher than in the captive macaques. The high abundance of Firmicutes was observed in other wildlife such as sika deer, and rhinoceros^62,63^. This might probably be the co-adaptation of microbiota and wild animals to enhance the efficacy of energy storage because wild animals can face the situation of starvation or survival in low food abundance. Supporting this hypothesis, the significant association between a greater Firmicutes/Bacteroidetes ratio and higher body fat in wild macaques was detected. Interestingly, the higher Firmicutes/Bacteroidetes ratio in oral microbiome of wild macaque was also observed. The ratio of Firmicutes/Bacteroidetes in oral microbiota was positively correlated with skinfold data similar to that in fecal microbiome. However, there was no previous study related to the association between Firmicutes/Bacteroidetes ratio in oral microbiota and body fat. Therefore, these results should be carefully interpreted. Further analysis of the relationship between bacteria in oral cavity and host metabolism may be helpful for better elucidating the functions of oral microbiota.

The inference of functional analysis showed the different activities of biochemical pathways in the microbiota of wild and captive macaques. The oral microbiota of captive macaques had a high preference for glycan metabolism such as glycosaminoglycans (GAGs) degradation pathway. A previous study reported that the GAGs degradation properties of bacteria could facilitate their binding to the host epithelial cells^64^. Therefore, most oral bacteria in captive macaques might have more potential to colonize and persist in the oral environment. In wild macaques, the oral microbiota was enriched with amino acid metabolism and lipid biosynthesis, but the sequential outcomes have not been elucidated.

Interestingly, the fecal microbiome of captive macaques increased the citrate cycle (tricarboxylic acid; TCA cycle) and glyoxylate and dicarboxylate metabolism which were suggested to reduce energy consumption and promote energy production^65^. Additionally, the increment of the TCA cycle could oxidize the SCFAs to generate ATP^66^. Therefore, this might cause a lower SCFA uptake into the host and limit energy storage. In contrast, the microbiota in the wild group were involved in ketone body metabolism. It has been reported that gut microbes could enhance ketone metabolism and use it as an energy source during starvation (low blood glucose) in humans^67^. This mechanism could also be the consequence of SCFA production for energy storage as suggested in a previous report^68^. Hence, the microbiota might play important roles in energy reserves and fat accumulation in wild macaques.

The findings of this study provided the preliminary overviews of microbiota in wild long-tailed macaques living in the natural habitat and wild macaques that were transferred from the natural habitat and reared in hygienic housing conditions in the captivity. This study can be conducted only in the countries where the distribution range of long-tailed macaques is and where the primate center in compliance with the national standard (such as AAALAC International) is established. As mentioned above, several captive macaque colonies bred in other primate centers, especially in the US, have usually been facing with the diarrhea problems which might be caused by microbiota dysbiosis^26,69^. From our field observation, we had not seen the diarrhea in wild animals. Besides, there was no comparison data of the oral-gut microbiota between wild animals living in natural habitat and that wild animals after transferred to hygienic captive condition and depicted how the oral-gut microbiota altered which can help to guide for the good practice of the animal care and management. The finding of this study would be useful for selection the beneficial bacteria for development of probiotics to mitigate the diarrhea in captive long-tailed macaques. For example, the bacteria of family Ruminococcaceae were enriched in wild macaque’s gut comparing to that of the captive animals (see Figs. 2, 3). It was suggested that these bacteria were butyrate-producer that may enhance anti-inflammation in the mucosal environment^57^. Thus, the future study is that the probiotics containing the bacteria complex of family Ruminococcaceae will be produced, fed to diarrhea captive long-tailed macaques, and observe if the symptom is resolved.

In summary, this study presented an overview of the effects of the captive environments on microbiome diversity and the abundance of wild-borne Thai long-tailed macaques. The findings implied that the changes of the environmental exposure and types of diet from the natural habitat to the captivity for only 1 year could alter oral-gut microbiota diversity and abundance in long-tailed macaques. The microbial profiles also suggested their putative metabolic functions. However, the interpretation should be cautious due to the lack of metagenomics and metabolomics data in this study. Moreover, the differences of origin and genetic background between the populations could also be a limitation of our study. Nevertheless, the information gained in this study represents a basis for functional microbiome research in long-tailed macaques, implementing the improvement of animal health care. The proper diet feeding program, or a novel probiotics development may be useful to modulate the microbiota protecting against dysbiosis and pathogen infections in these animals in the captive environment.

## Methods

### Animal experiment statement

All methods were performed in accordance with the relevant guidelines and regulations. The animal use protocol was examined and approved by the committee of animal care and use at NPRCT-CU (Protocol Review No. 1775005). Study design and animal specimen collection were carried out and reported following the ARRIVE guidelines (https://arriveguidelines.org/arrive-guidelines).

### Animal cohort

Two groups of 80 male and female long-tailed macaques (*Macaca fascicularis*); wild (*n* = 37) and captive (*n* = 43) at the age between 1.5 and 18 years were recruited for this study. The age and body weights of each group of animals were presented in Table 1. The ages of animals were estimated based on the dental eruption pattern established by Smith et al.^70^. The wild macaques inhabited the Wat Tham Praporthisat, central Thailand. The captive macaques were wild borne at Khaoson-Samae Dam, central Thailand, captured, and translocated to NPRCT-CU for 1 year before the date of sample collection. They were socially housed in strictly hygienic condition. They were housed in semi-opened gang cages (4 × 4 × 3; W × L × H), supplied with hyperchlorinated water (1 ppm) through automatic Lixit, and fed two times a day. The food provisioning program at NPRCT-CU is the standard monkey pellets (Perfect Companion Group Co., Ltd, Thailand) during 9–10 AM and fresh fruits during (2–3 PM). The fresh fruits were, for example, bananas, watermelons, melons, dragon fruits, pineapples, and oranges. The facility has been AAALAC International accreditation. The captive macaque did not received antibiotic, treatments, and not in use for any experiment. The wild macaque capture was approved from the Department of National Parks, Wildlife and Plant Conservation; permission no. 0909.702/1431 (25 Jan 2016) and 0909.302/5369 (25 Mar 2014).

### Sample collections

The fecal and oral swabs collection of wild macaques was performed in the field site, while that of the captive (wild-born) macaques was performed during the annual health check. The specimens were collected when the macaques were anesthetized with a mixture of dexmedetomidine hydrochloride (Zoetis, USA) (0.03–0.05 mg/kg) and Zoletil (Virbac, New Zealand) (3–5 mg/kg). The polyester tipped swabs (Puritan, USA) was used for specimen collection performed by experienced veterinarians. The oral swab of each individual was obtained by swabbing on both sides of buccal surface as well as lower and upper gum. The fecal specimen was collected by rectal swab directly from animals. The collected swabs were transferred into 3 mL of viral transport media which was fleshly prepared mixtures comprised of Hank’s balanced salt solution (Gibco, ThermoFisher Scientific, USA), 1% (w/v) of bovine serum albumin (Gibco, ThermoFisher Scientific, USA), 100 U/mL of penicillin G (Calbiochem, Merck, Germany), 50 μg/mL of streptomycin (Calbiochem, Merck, Germany), and 15 μg/mL of amphotericin B (Supelco, Merck, Germany). The collected samples stored at 4 °C, transferred to the laboratory within 12 h. Then, they were aliquot and kept at − 80 °C until used. In addition, the skinfold measurement was also conducted to record the body fat accumulation in these macaques following the procedure described in previous study^71^.

### DNA preparation and 16S microbiome sequencing

Total DNA extraction was performed as previously described in Sawaswong et al.^37^ The V4 region of the bacterial 16S small subunit ribosomal RNA (16S rRNA) gene was amplified based on specific primers 16S-515F (5′-GTGCCAGCMGCCGCGGTAA-3′) and 16S-806R (5′-GGACTACHVGGGTWTCTAAT-3′) containing phasing sequences and TruSeq adaptor modified from previous study^72^. The 20 µL PCR reaction contained 10 ng of DNA template, 0.2 µM of each primer, 0.2 mM of dNTPs, and 0.4 U of Phusion DNA Polymerase (Thermo Scientific, USA). The PCR reaction was then amplified following thermal condition: 98 °C for 30 s; 25 cycles of 98 °C for 10 s, 55 °C for 25 s, 72 °C for 25 s; 72 °C for 10 min. After that, the product was then performed second round PCR to attach the multiplexing index and adaptors as described previously^37^. Finally, the library was paired-end (2 × 250) sequenced by the Illumina MiSeq platform (Illumina, USA).

### Data analysis

Raw data were demultiplexed by MiSeq reporter software (version 2.6.2.3). The FASTQ sequences were analyzed using the QIIME2 pipeline (version 2018.8). Briefly, the mate paired reads were joined and quality trimmed (> Q30). The deduplication and de-novo clustering (97% similarity) were performed by the VSEARCH algorithm. The chimeric sequences were eliminated by the UCHIME algorithm. Sequences of OTUs were then classified by comparing against Greengenes bacterial rRNA database (version 13.8) using VSEARCH. Differential abundance comparison of bacteria between captive and wild macaques was investigated by Linear discriminant analysis effect size (LEfSe). The significantly different taxa (*P* < 0.01) with Linear discriminant analysis (LDA) score > 3 were selected to plot the cladogram. The functional inference of bacterial microbiome was analyzed using the Phylogenetic Investigation of Communities by Reconstruction of Unobserved States (PICRUSt2) software. The KEGG ortholog (KO)^73^ abundances of wild and captive macaques were statistically tested by multiple *t*-tests with 1% false discovery rate (FDR) correction.

## Supplementary Information

Supplementary Information.

## Data Availability

The datasets generated from next-generation sequencing during the current study are available in the NCBI Sequence Read Archive (SRA) repository, BioProject ID: PRJNA705799.

## References

[1] Cani PD (2018). Human gut microbiome: Hopes, threats and promises. Gut.

[2] Hanning I, Diaz-Sanchez S (2015). The functionality of the gastrointestinal microbiome in non-human animals. Microbiome.

[3] Valdes AM, Walter J, Segal E, Spector TD (2018). Role of the gut microbiota in nutrition and health. BMJ.

[4] Jandhyala SM (2015). Role of the normal gut microbiota. World J. Gastroenterol..

[5] Chambers ES, Preston T, Frost G, Morrison DJ (2018). Role of gut microbiota-generated short-chain fatty acids in metabolic and cardiovascular health. Curr. Nutr. Rep..

[6] Turnbaugh PJ (2006). An obesity-associated gut microbiome with increased capacity for energy harvest. Nature.

[7] Evron R, Polacheck I, Guizie M, Levy M, Zehavi U (1988). Activities of compound G2 isolated from alfalfa roots against dermatophytes. Antimicrob. Agents Chemother..

[8] Hold GL (2014). Role of the gut microbiota in inflammatory bowel disease pathogenesis: What have we learnt in the past 10 years?. World J. Gastroenterol..

[9] Cani PD, Jordan BF (2018). Gut microbiota-mediated inflammation in obesity: A link with gastrointestinal cancer. Nat. Rev. Gastroenterol. Hepatol..

[10] Rothschild D (2018). Environment dominates over host genetics in shaping human gut microbiota. Nature.

[11] Chen Z (2018). Diversity of macaque microbiota compared to the human counterparts. Sci. Rep..

[12] Hillman ET, Lu H, Yao T, Nakatsu CH (2017). Microbial ecology along the gastrointestinal tract. Microbes Environ..

[13] Deo PN, Deshmukh R (2019). Oral microbiome: Unveiling the fundamentals. J. Oral Maxillofac. Pathol..

[14] Dantas G, Sommer MO, Degnan PH, Goodman AL (2013). Experimental approaches for defining functional roles of microbes in the human gut. Annu. Rev. Microbiol..

[15] Nagpal R (2018). Comparative microbiome signatures and short-chain fatty acids in mouse, rat, non-human primate, and human feces. Front. Microbiol..

[16] Rivera-Hernandez T (2014). The contribution of non-human primate models to the development of human vaccines. Discov. Med..

[17] Pryor R, Martinez-Martinez D, Quintaneiro L, Cabreiro F (2020). The role of the microbiome in drug response. Annu. Rev. Pharmacol. Toxicol..

[18] Uno Y, Uehara S, Yamazaki H (2016). Utility of non-human primates in drug development: Comparison of non-human primate and human drug-metabolizing cytochrome P450 enzymes. Biochem. Pharmacol..

[19] Ciabattini A, Olivieri R, Lazzeri E, Medaglini D (2019). Role of the microbiota in the modulation of vaccine immune responses. Front. Microbiol..

[20] Kyes RC (1993). Survey of the long-tailed macaques introduced onto Tinjil Island, Indonesia. Am. J. Primatol..

[21] Guebre-Xabier M (2020). NVX-CoV2373 vaccine protects cynomolgus macaque upper and lower airways against SARS-CoV-2 challenge. Vaccine.

[22] Fooden J (1997). Tail length variation in *Macaca fascicularis* and *M. mulatta*. Primates.

[23] Malaivijitnond S, Hamada Y (2008). Current situation and status of long-tailed macaques (*Macaca fascicularis*) in Thailand. Trop. Nat. Hist..

[24] McKenzie VJ (2017). The effects of captivity on the mammalian gut microbiome. Integr. Comp. Biol..

[25] Rosshart SP (2017). Wild mouse gut microbiota promotes host fitness and improves disease resistance. Cell.

[26] Koo BS (2020). Idiopathic chronic diarrhea associated with dysbiosis in a captive cynomolgus macaque (*Macaca fascicularis*). J. Med. Primatol..

[27] Bolyen E (2019). Reproducible, interactive, scalable and extensible microbiome data science using QIIME 2. Nat. Biotechnol..

[28] Segata N (2011). Metagenomic biomarker discovery and explanation. Genome Biol..

[29] Douglas GM (2020). PICRUSt2 for prediction of metagenome functions. Nat. Biotechnol..

[30] Chattopadhyay I, Verma M, Panda M (2019). Role of oral microbiome signatures in diagnosis and prognosis of oral cancer. Technol. Cancer Res. Treat..

[31] Abranches J (2018). Biology of oral streptococci. Microbiol. Spectr..

[32] Krishnan K, Chen T, Paster BJ (2017). A practical guide to the oral microbiome and its relation to health and disease. Oral Dis..

[33] Ozga AT (2019). Oral microbiome diversity in chimpanzees from Gombe National Park. Sci. Rep..

[34] Elliott DR, Wilson M, Buckley CM, Spratt DA (2005). Cultivable oral microbiota of domestic dogs. J. Clin. Microbiol..

[35] Deeb BJ, Quesenberry KE, Carpenter JW (2004). Ferrets, rabbits, and rodents. Respiratory Disease and Pasteurellosis.

[36] Li X (2020). The microbiome of captive hamadryas baboons. Anim. Microbiome.

[37] Sawaswong V (2020). Oral-fecal mycobiome in wild and captive cynomolgus macaques (*Macaca fascicularis*). Fungal Genet. Biol..

[38] Daims H, Wagner M (2018). Nitrospira. Trends Microbiol..

[39] Singh S (2020). Gemmata obscuriglobus: A connecting link between prokaryotic and eukaryotic cell. Biologia.

[40] Wolińska A, Das S, Dash HR (2019). Metagenomic achievements in microbial diversity determination in croplands. Microbial Diversity in the Genomic Era.

[41] Beeckmans S, Xie JP (2015). Reference Module in Biomedical Sciences.

[42] Rosenberg E (2014). The Prokaryotes: Alphaproteobacteria and Betaproteobacteria.

[43] Kasalicky V, Jezbera J, Hahn MW, Simek K (2013). The diversity of the Limnohabitans genus, an important group of freshwater bacterioplankton, by characterization of 35 isolated strains. PLoS ONE.

[44] Kim S, Covington A, Pamer EG (2017). The intestinal microbiota: Antibiotics, colonization resistance, and enteric pathogens. Immunol. Rev..

[45] Clayton JB (2016). Captivity humanizes the primate microbiome. Proc. Natl. Acad. Sci. U.S.A..

[46] Nishida AH, Ochman H (2019). A great-ape view of the gut microbiome. Nat. Rev. Genet..

[47] Arumugam M (2011). Enterotypes of the human gut microbiome. Nature.

[48] Simpson HL, Campbell BJ (2015). Review article: Dietary fibre-microbiota interactions. Aliment Pharmacol. Ther..

[49] Iljazovic A (2021). Perturbation of the gut microbiome by *Prevotella* spp. enhances host susceptibility to mucosal inflammation. Mucosal Immunol..

[50] De Filippo C (2017). Diet, environments, and gut microbiota. A preliminary investigation in children living in rural and urban Burkina Faso and Italy. Front. Microbiol..

[51] Allan N (2018). Conservation implications of shifting gut microbiomes in captive-reared endangered voles intended for reintroduction into the wild. Microorganisms.

[52] Kohl KD, Sadowska ET, Rudolf AM, Dearing MD, Koteja P (2016). Experimental evolution on a wild mammal species results in modifications of gut microbial communities. Front. Microbiol..

[53] Niu Q (2015). Dynamic distribution of the gut microbiota and the relationship with apparent crude fiber digestibility and growth stages in pigs. Sci. Rep..

[54] Tokuda G (2018). Fiber-associated spirochetes are major agents of hemicellulose degradation in the hindgut of wood-feeding higher termites. Proc. Natl. Acad. Sci. U.S.A..

[55] Xie X (2018). Persistence of cellulolytic bacteria fibrobacter and treponema after short-term corn stover-based dietary intervention reveals the potential to improve rumen fibrolytic function. Front. Microbiol..

[56] Biddle A, Stewart L, Blanchard J, Leschine S (2013). Untangling the genetic basis of fibrolytic specialization by lachnospiraceae and ruminococcaceae in diverse gut communities. Diversity.

[57] Graham C, Mullen A, Whelan K (2015). Obesity and the gastrointestinal microbiota: A review of associations and mechanisms. Nutr. Rev..

[58] Cao Y, Shen J, Ran ZH (2014). Association between *Faecalibacterium prausnitzii* reduction and Inflammatory Bowel Disease: A meta-analysis and systematic review of the literature. Gastroenterol. Res. Pract..

[59] Aguirre M, Jonkers DM, Troost FJ, Roeselers G, Venema K (2014). In vitro characterization of the impact of different substrates on metabolite production, energy extraction and composition of gut microbiota from lean and obese subjects. PLoS ONE.

[60] Turnbaugh PJ (2009). The effect of diet on the human gut microbiome: A metagenomic analysis in humanized gnotobiotic mice. Sci. Transl. Med..

[61] Turnbaugh PJ, Gordon JI (2009). The core gut microbiome, energy balance and obesity. J. Physiol..

[62] Guan Y (2017). Comparison of the gut microbiota composition between wild and captive sika deer (*Cervus nippon* hortulorum) from feces by high-throughput sequencing. AMB Express.

[63] Gibson KM (2019). Gut microbiome differences between wild and captive black rhinoceros—Implications for rhino health. Sci. Rep..

[64] Kawai K, Kamochi R, Oiki S, Murata K, Hashimoto W (2018). Probiotics in human gut microbiota can degrade host glycosaminoglycans. Sci. Rep..

[65] Yang Y (2020). Caloric restriction remodels energy metabolic pathways of gut microbiota and promotes host autophagy. BioRxiv..

[66] Selkrig J, Wong P, Zhang X, Pettersson S (2014). Metabolic tinkering by the gut microbiome: Implications for brain development and function. Gut Microbes.

[67] Crawford PA (2009). Regulation of myocardial ketone body metabolism by the gut microbiota during nutrient deprivation. Proc. Natl. Acad. Sci. U.S.A..

[68] Mestdagh R (2012). Gut microbiota modulate the metabolism of brown adipose tissue in mice. J. Proteome Res..

[69] Clayton JB, Danzeisen JL, Trent AM, Murphy T, Johnson TJ (2014). Longitudinal characterization of *Escherichia coli* in healthy captive non-human primates. Front. Vet. Sci..

[70] HollySmith B, Crummett TL, Brandt KL (1994). Ages of eruption of primate teeth: A compendium for aging individuals and comparing life histories. Am. J. Phys. Anthropol..

[71] Hamada Y, Suryobroto B, Goto S, Malaivijitnond S (2008). Morphological and body color variation in Thai *Macaca fascicularis* fascicularis North and South of the Isthmus of Kra. Int. J. Primatol..

[72] Wu L (2015). Phasing amplicon sequencing on Illumina Miseq for robust environmental microbial community analysis. BMC Microbiol..

[73] Kanehisa M, Sato Y, Kawashima M, Furumichi M, Tanabe M (2016). KEGG as a reference resource for gene and protein annotation. Nucleic Acids Res..

