# Supplementary material for: Comparative analysis of oral-gut microbiota between captive and wild long-tailed macaque in Thailand
Source: Sci Rep. 2021 Jul 12;11:14280. doi: 10.1038/s41598-021-93779-4 (PMC8275770; doi:10.1038/s41598-021-93779-4)
Supplement: Supplementary file 1 — Supplementary Information. [file 41598_2021_93779_MOESM1_ESM.pdf]

Comparative analysis of oral-gut microbiota between captive and wild long-tailed macaque in Thailand

Vorthon Sawaswong [1,2], Kesmanee Praianantathavorn [3], Prangwalai Chanchaem [2], Ariya Khamwut [2], Taratorn Kemthong [4], Yuzuru Hamada [5], Suchinda Malaivijitnond [4,6], Sunchai Payungporn [2,3]

1 Program in Bioinformatics and Computational Biology, Graduate School, Chulalongkorn University, Bangkok 10330, Thailand

2 Research Unit of Systems Microbiology, Chulalongkorn University, Bangkok 10330, Thailand

3 Department of Biochemistry, Faculty of Medicine, Chulalongkorn University, Bangkok 10330, Thailand

4 National Primate Research Center of Thailand, Chulalongkorn University, Saraburi 18110, Thailand

5 Evolutionary Morphology Section, Primate Research Institute, Kyoto University, Aichi, Japan

6 Department of Biology, Faculty of Science, Chulalongkorn University, Bangkok 10330, Thailand

* Correspondence: sp.medbiochemcu@gmail.com

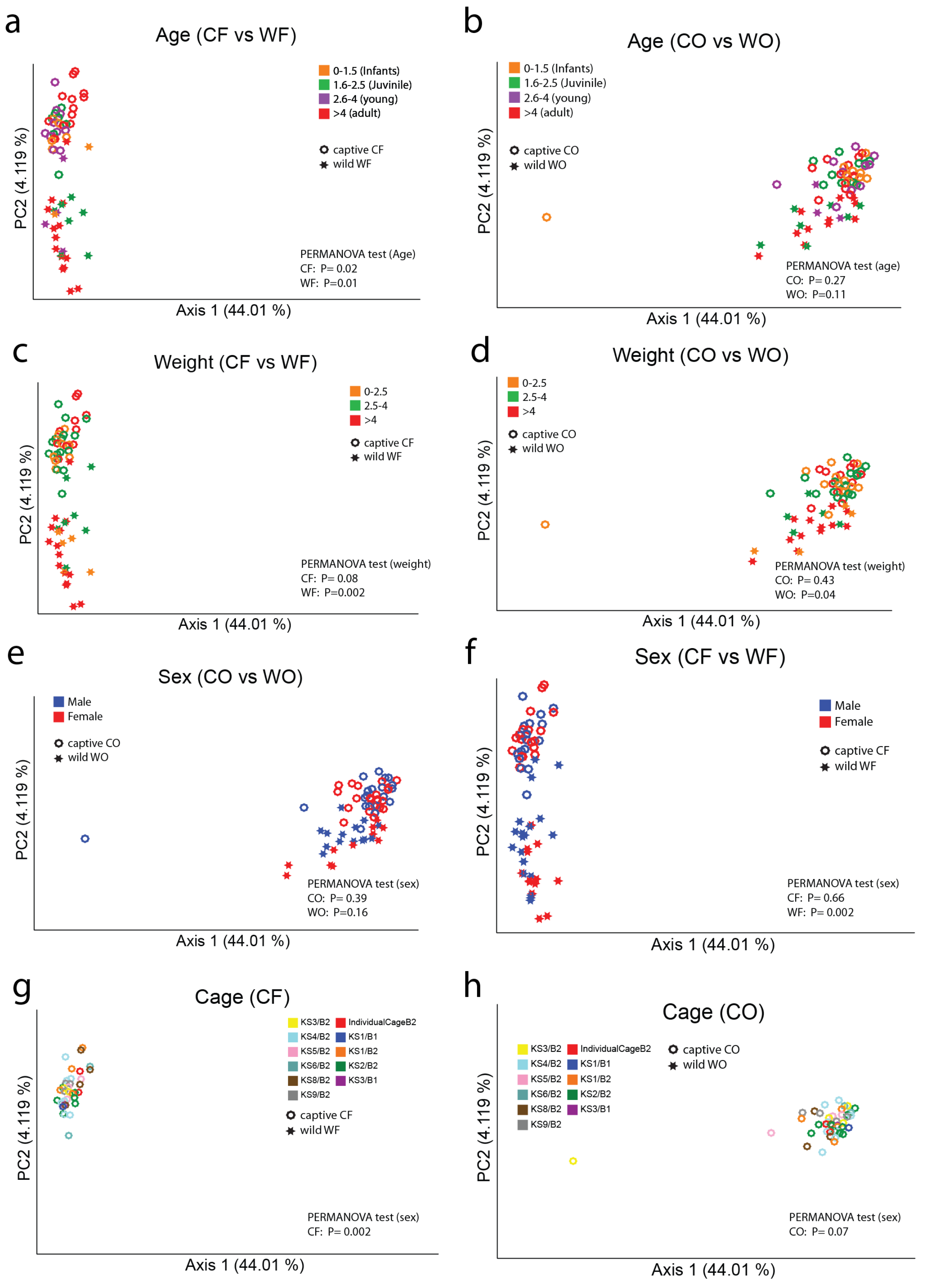

Supplementary Figure S1. The beta diversity was plotted in principal coordinate analysis (PCoA) plots based on Jaccard dissimilarity. The PCoA plots were labelled by factor including **a.** age (CF vs WF), **b.** age (CO vs WO), **c.** weight (CF vs WF), **d.** weight (CO vs WO), **e.** sex (CF vs WF), **f.** sex (CO vs WO), **g.** cage (CF), and **h.** cage (CO).

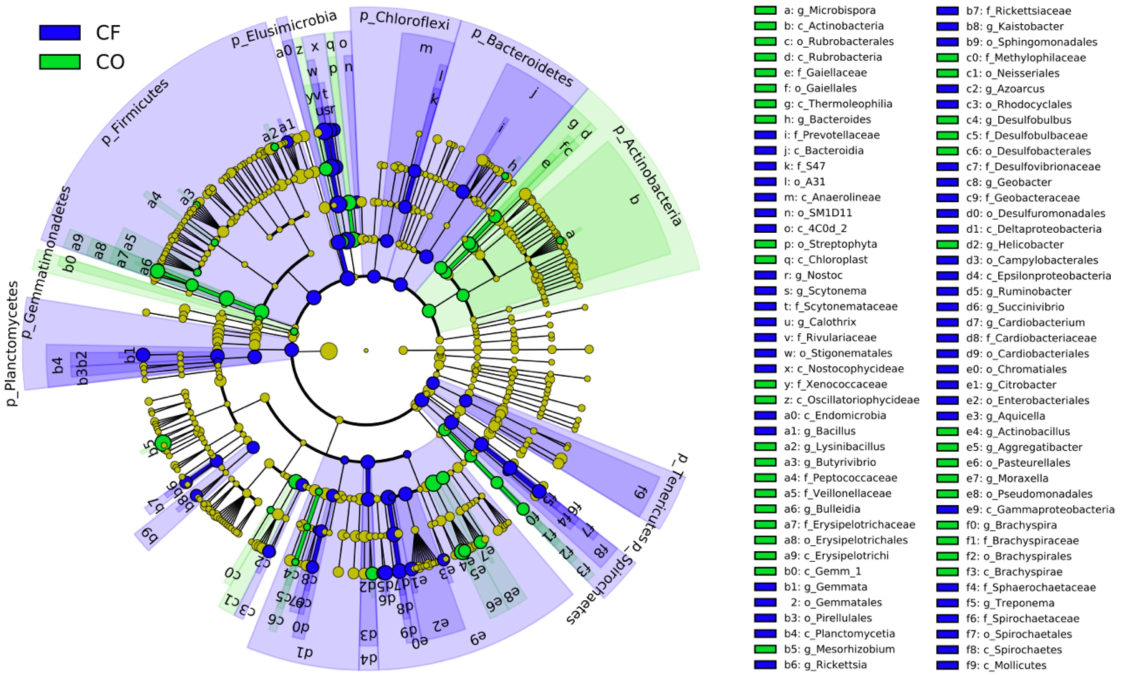

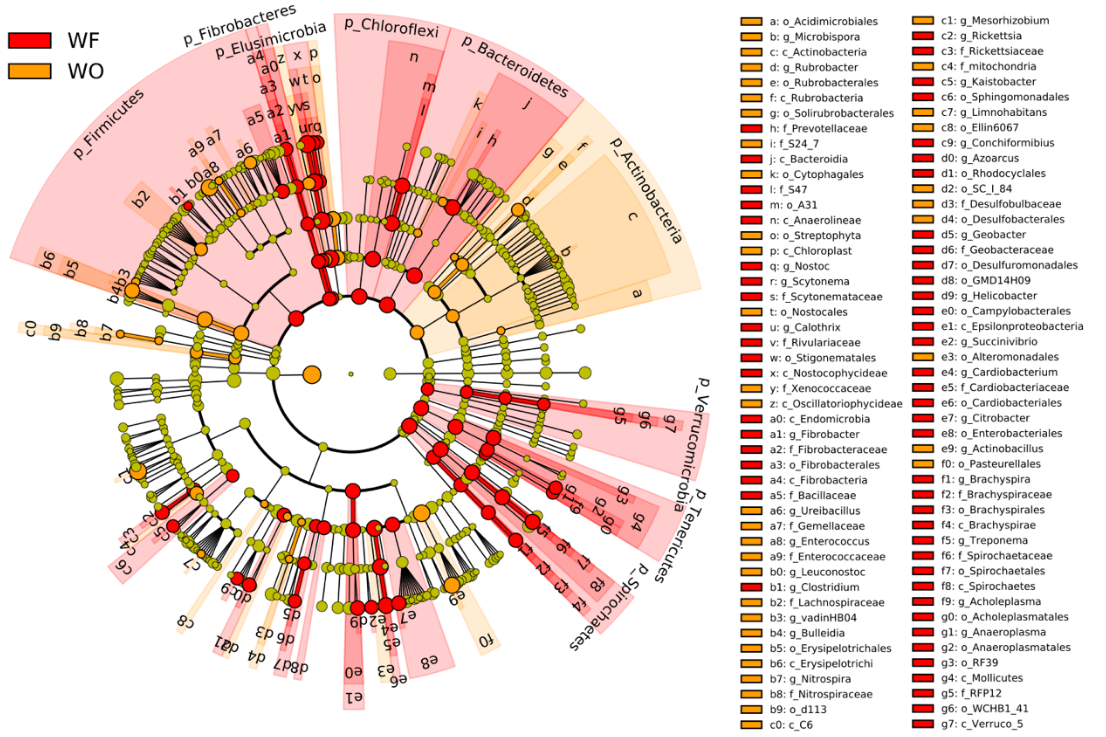

Supplementary Figure S2. Differential abundance analysis comparing the taxa within oral and fecal microbiome of cynomolgus macaques. The cladogram illustrated the results from Linear discriminant analysis Effect Size (LEfSe) analysis showing the significantly differential taxa (LDA>3, *P*<0.05) in **a** captive macaque (CO vs CF) and **b** wild macaque (WO vs WF).

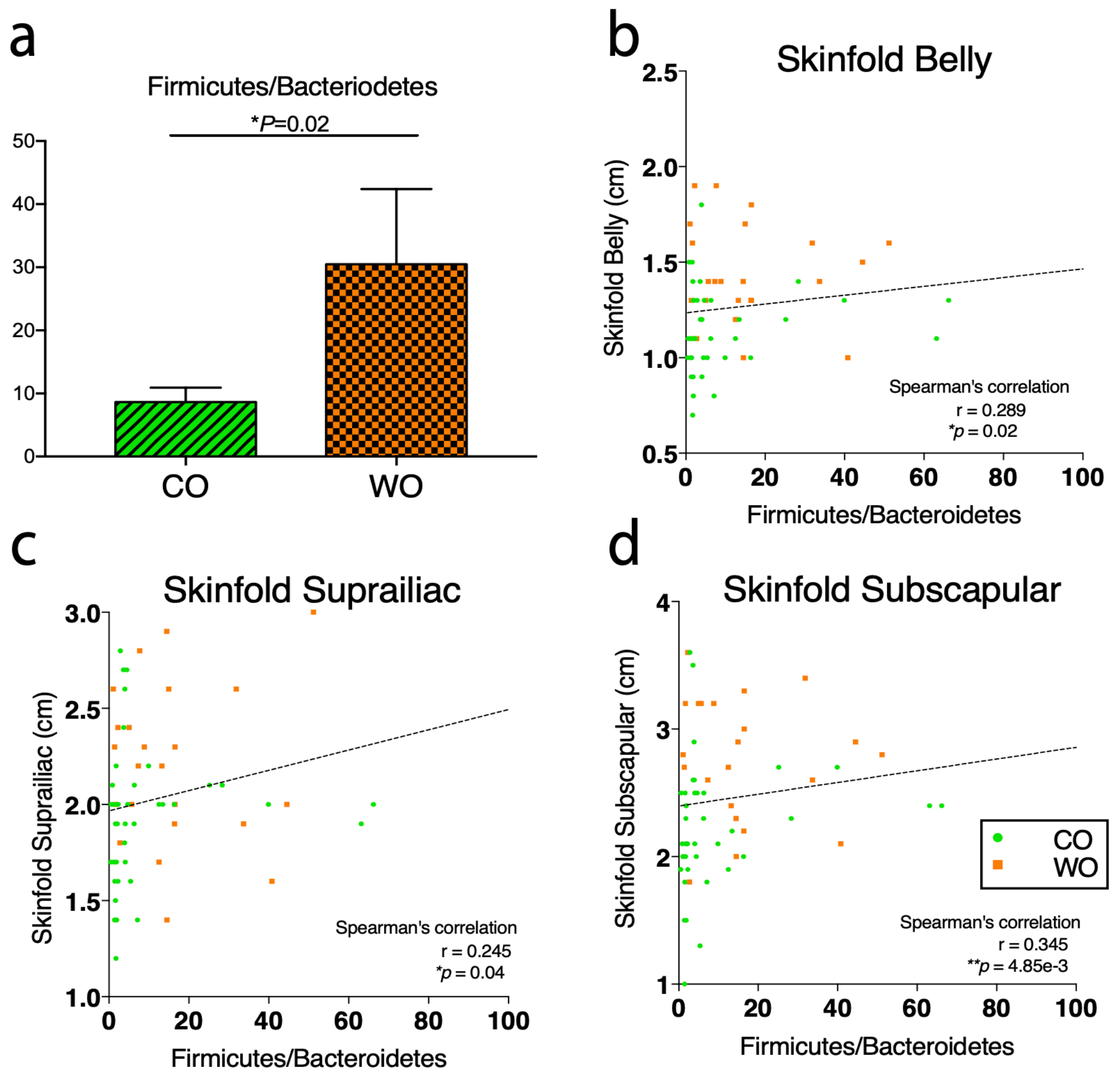

Supplementary Figure S3. The association of Firmicutes/Bacteroidetes ratio in oral microbiome and body fat accumulations in cynomolgus macaque. **a** The difference of Firmicutes/Bacteroidetes ratio (mean ± SEM) between wild and captive was compared by *t*-test. (**P*<0.05, ***P*<0.01) The scatter dot plot presents the correlation between Firmicutes/Bacteroidetes and **b** skinfold belly, **c** skinfold suprailiac, and **d** skinfold subscapular analyzed by Spearman's correlation test.
